# Expression of small breast epithelial mucin (SBEM) protein in tissue microarrays (TMAs) of primary invasive breast cancers

**DOI:** 10.1111/j.1365-2559.2007.02955.x

**Published:** 2008-02

**Authors:** G P Skliris, F Hubé, I Gheorghiu, M M Mutawe, C Penner, P H Watson, L C Murphy, E Leygue, Y Myal

**Affiliations:** 1 Department of Biochemistry and Medical Genetics, University of Manitoba Winnipeg, Manitoba, Canada; 2 Manitoba Institute of Cell Biology, University of Manitoba Winnipeg, Manitoba, Canada; 3 Department of Physiology, University of Manitoba Winnipeg, Manitoba, Canada; 4 Department of Pathology, University of Manitoba Winnipeg, Manitoba, Canada

**Keywords:** breast cancer, immunohistochemistry, small breast epithelial mucin, survival analysis, tissue microarrays, tumour biomarker

## Abstract

**Aims:**

Small breast epithelial mucin (SBEM) is a recently described gene product that shows promise as a new breast biomarker. The aim was to investigate for the first time SBEM protein expression in a large cohort (*n* = 300) of invasive breast cancers, its relationship to established clinical variables and its association with clinical outcome.

**Methods and results:**

Immunohistochemical analysis was performed on tissue microarrays consisting of 149 oestrogen receptor (ER) α− and 151 ERα+ breast cancers. Overall, 18% of tumours were SBEM+ (*n* = 53/300). However, SBEM protein was more frequently observed in ER− (22%) than in ER+ cancers (13%; *P* = 0.049). A significant association with psoriasin/S100A7 expression (*P*≤ 0.0001) was observed in the entire cohort. SBEM was also positively associated with HER-2 (*P* = 0.046) in ER− cancers, and increased levels of SBEM were strongly associated with higher tumour grade (*P* = 0.0015). Furthermore, SBEM expression showed a trend towards an association with reduced overall survival and relapse-free survival in the ER+ cohort (*P* = 0.063 and *P* = 0.072, respectively).

**Conclusions:**

Our results suggest that SBEM may identify a unique subset of breast cancers with poor prognosis and may have future implications for therapeutic management of this disease.

## Introduction

Breast cancer is the most common cancer among women and is the second leading cause of cancer death in women in developed countries.^1^,^2^ The 5-year survival rate is approximately 75% for women with locally advanced breast cancer.^2^ However, if the cancer has metastasized, the average survival time falls to <2 years,^3^,^4^ and at such time a very limited number of treatment options are available for patient management.

To date, oestrogen receptor (ER) and progesterone receptor (PR) are the two most useful clinical markers predicting response to hormonal therapy in both metastatic disease and in the adjuvant setting.^5^–^7^ More recently, HER*-*2, which is overexpressed in 25% of breast cancers and is associated with aggressive disease and poor prognosis, has also been used in the clinical setting, as a prognostic and predictive marker.^8^ Many other markers have been or are being investigated, including the mucin 1 (*MUC1*) gene product and related tumour antigens (CA27.29), carcinogenic embryonic antigen, cathepsin D, gross cystic disease fluid protein/human prolactin inducible protein, mammaglobin and p53.^9^–^15^ Because breast cancer encompasses a highly heterogeneous group of tumours, our failure to treat or cure a given patient may result from our inability to match the particular phenotype of the tumour cells of the patient with the appropriate treatment. Therefore, there is an urgent need to identify and evaluate new breast biomarkers for monitoring both progression and treatment of the disease. Prognostically significant clusters of breast cancers have recently been identified by a number of investigators reporting on gene profiling and tissue microarray (TMA) analyses. The biologically distinct groups of markers defining these tumour clusters are likely to have real clinical and prognostic/diagnostic relevance in the near future.^16^–^18^ Identification of tumour markers with expression that is restricted to the mammary epithelium would have a significant impact not only on reporting proliferative changes in the breast (which may predict increased risk of breast cancer development), but also on enhancing the detection of micrometastatic disease by identifying cancer cells of breast origin in lymph nodes.

The small breast epithelial mucin (*SBEM*) gene, also known as BS106 and/or B511S, was originally identified as a putative breast-specific gene.^19^–^22^*SBEM* encodes a secreted 90 amino acids glycoprotein, which consists of a secretion signal peptide, three tandemly repeated octapeptide motifs (TTAAXTTA) and exhibits characteristics of members of the mucin family.^20^ Dot blot analysis has revealed that *SBEM* RNA is highly expressed in normal breast and salivary glands, but not in other normal tissues tested, including prostate, lung, ovary and testis.^19^,^20^ Similarly, reverse transcriptase-polymerase chain reaction (RT-PCR) analysis of cDNA extracted from various cancer cell lines has revealed consistently high levels of *SBEM* in breast cancer cell lines (7/8), whereas none of the six non-mammary cell lines tested was positive.^19^,^20^ Furthermore, *SBEM* gene expression, as assessed by RT-PCR, has been observed in >90% of primary or metastatic breast cancers.^19^,^20^ Moreover, it has recently been demonstrated that the combined use of SBEM, cytokeratin (CK) 19, trefoil factor-3 (p1B) and epithelial glycoprotein-2 (EGP-2) expression allows the identification of micrometastases in sentinel node biopsy specimens of breast cancers, missed by standard histological evaluation.^23^

Overall, the accumulated data so far suggest that the utility of SBEM as a new breast cancer marker may be promising. However, with the exception of one study,^19^ which was carried out on a very small cohort of breast cancers, all studies conducted to date have examined *SBEM* mRNA expression. In the latter study an increase of SBEM protein expression in tumours versus normal breast tissue was observed. To date, there have been no reports in which large cohorts of selected breast cancer cases have been evaluated for SBEM protein expression and in which differences in SBEM protein expression by subject characteristics have been examined. In the present study, we have investigated SBEM protein expression by immunohistochemistry (IHC) in TMAs and its association with other established markers of prognosis, in a large cohort of invasive breast cancers corresponding to 300 patients.

## Materials and methods

### Breast tumour tissue microarrays

All invasive breast cancers used in the current study were obtained from the Manitoba Breast Tumour Bank (MBTB, Department of Pathology, University of Manitoba), which operates with the approval of the Faculty of Medicine, University of Manitoba, Research Ethics Board.^24^ As described previously, all tissues are accrued to the bank from cases at multiple centres within Manitoba, which are collected and frozen at −70°C immediately after surgical removal. A portion of the frozen tissue from each case is then processed to create matched formalin-fixed paraffin-embedded and frozen tissue blocks. The histopathology of all MBTB cases has been previously assessed and entered into a computerized database to enable selection based on composition of the tissue as well as clinicopathological parameters. After selection, cases were re-reviewed on haematoxylin and eosin sections by a breast pathologist (P.H.W.). ER+ and ER− TMAs were constructed from cohorts of 246 ERα+ and 255 ERα− primary invasive ductal breast carcinomas, respectively. In brief, duplicate core tissue samples (0.6 mm diameter) were taken from selected areas of maximum cellularity for each tumour with a tissue arrayer instrument (Beecher Instruments, Silver Spring, MD, USA). Only tumour biopsy specimens whose ER status was determined both by ligand binding assay (LBA; ER+ >3 fmol/mg protein) and by IHC were included. Ultimately, 300 cases provided reliable information. In addition, a commercially available TMA (Biomax Inc., Rockville, MD, USA; cat. No. BN08013, http://www.biomax.us) consisting of 68 normal breast tissue biopsy specimens was also analysed by IHC.

### Clinicopathological characteristics of patient cohorts

The recommendations for tumour marker prognostic studies (REMARK) as reported by McShane^25^ were followed as closely as possible. Case selection was based on the following criteria: (i) a minimum patient follow-up of 60 months; (ii) tumours had an invasive component of >20% of the tissue section, and ≤10% of the normal epithelial content; (iii) ER− status was defined by LBA criteria of ≤3 fmol/mg protein. The criteria for interpretation of the variables were as follows: (i) PR+ status was defined as >10 fmol/mg protein by LBA; (ii) tumour grading was consistent with the Nottingham system (scores 3–5 = low; 6–7 = moderate; and 8–9 = high); (iii) tumour size was classified as either small (≤20 mm) or large (>20 mm); (iv) tumour inflammation was assessed on a scale from 1 to 5 and then assigned to low (score 1–3) or high (score 4 and 5) categories. In the ER− TMA (*n* = 149), patients received a variety of treatments, such as hormonal therapy (*n* = 21), hormonal plus radiotherapy (*n* = 6), hormonal, radiotherapy and chemotherapy (*n* = 13), hormonal plus chemotherapy (*n* = 4), radiotherapy alone (*n* = 5), radiotherapy plus chemotherapy (*n* = 43), chemotherapy alone (*n* = 32), surgery alone (*n* = 21), and for four patients the treatment regimen was unknown. In the ER+ TMA (*n* = 151), patients received the following treatments: hormonal therapy alone (*n* = 92), hormonal plus radiotherapy (*n* = 56), hormonal and chemotherapy (*n* = 1) and radiotherapy alone (*n* = 1), whereas one patient had surgery alone (*n* = 1).

### Breast cancer sections

Ten pairs of matched frozen and paraffin-embedded breast cancers were selected from the MBTB for examining SBEM gene and protein expression. Serial tissue sections obtained from these biopsy specimens were evaluated for gene expression by both RT-PCR and Northern blot analysis, whereas protein expression was assessed by IHC and Western blot analysis. ER and PR levels ranged from 2.3 to 180 fmol/mg protein and 4.5 to 105 fmol/mg protein, respectively (nine ER+ and eight PR+ breast cancers). The ages of patients were between 58 and 78 years, and tumour size varied from 15 to 50 mm.

### RNA and protein extraction

Total RNA was extracted from 20-μm frozen tissue sections (10–20 sections/tumour) using the Trizol® RNA extraction protocol (Trizol®; Invitrogen, Carlsbad, CA, USA) according to the manufacturer’s instructions. Protein extraction was carried out as previously described.^26^ Briefly, tissues were homogenized in ice-cold homogenization buffer containing 20 mm 3-(*N*-morpholino)-propanesulfonic acid (pH 7.2–7.5), 60 mmβ-glycerophosphate, 5 mm ethylene glycol-bis (β-aminoethyl ether)-*N*,*N*,*N*′,*N*′-tetraaceticacid (pH 8.0), 5 mm sodium fluorate, 1 mm sodium vanadate, 1% NP40 (all from Sigma, St Louis, MO, USA) and a mini-protease inhibitor cocktail tablet (Boehringer Mannheim, Indianapolis, IN, USA) per 10-ml extraction buffer and sonicated several times using an ultrasonic cell disrupter (Sonics & Materials, Inc., Danbury, CT, USA). Sonicates were centrifuged at 13 000 ***g*** for 20 min at 4°C and stored at −20°C until use. Protein concentration was determined using the Lowry assay.^27^

### RT-PCR analysis

Two micrograms of total RNA was reverse-transcribed for 1 h at 37°C as previously described.^12^ A negative control for the RT-PCR was included in which no Moloney murine leukaemia virus (MMLV) enzyme was added. *SBEM* primers consisted of a forward primer designated *SBEM*-U (5′-gatcttcaggtcaccaccatg-3′) and a reverse primer designated *SBEM*-L (5′-gggacacactctaccattcg-3′). Two microlitres of each reverse transcription mixture, in the presence of 20 mm Tris–HCl (pH 8.4), 50 mm KCl, 1.5 mm MgCl_2_, 200 μm of each dNTP, 200 ng of each primer and 0.5 U of Taq DNA polymerase, was used for PCR in a final volume of 50 μl. Primers for glyceraldehyde-3-phosphate dehydrogenase (*GAPDH*) consisted of a forward primer *GAP*-U (5′-acccactcctccacctttg-3′) and a reverse primer *GAP*-L (5′-ctcttgtgctcttgctggg-3′). PCR products were visualized with UV irradiation on a GelDoc2000/ChemiDoc System (BioRad, Hercules, CA, USA) and quantified by densitometry using QuantityOne software (version 4.2; BioRad). Three independent PCRs (35 cycles; 15 s at 94°C, 15 s at 52°C and 15 s at 72°C) were performed from each separate RT reaction and the average of the relative signals was calculated. Briefly, *SBEM* signals in breast tumour no. 4 were arbitrarily set as 1.0 and *SBEM* signals in all other samples were quantified relative to tumour no. 4. This strategy was also employed for the quantification of the *GAPDH* (control gene, Table 2). All *SBEM* signals were expressed relative to the signal of tumour sample no. 4 (quantification of *SBEM* mRNA levels SEM; Table 2).

**Table 2:** Correlation of SBEM gene and protein expression in the small panel of 10 breast cancers

Breast cancer no.	Northern blot analysis	RT-PCRs *SBEM*/*GAPDH* signal ± SEM	IHC (H-score)	Western blot analysis
1	−	0.71 ± 0.13	0	−
2	+	1.52 ± 0.26	10	−
3	−	0.70 ± 0.17	5	−
4	−	1 ± 0	0	−
5	+	5.8 ± 1.55	150	+
6	+	1.92 ± 0.43	0	−
7	+	3.22 ± 1.26	10	−
8	+	0.90 ± 0.36	10	−
9	−	3.12 ± 1.50	20	−
10	+	4.56 ± 1.12	40	−

*SBEM* gene expression was primarily assessed by Northern blotting and secondarily by reverse transcriptase-polymerase chain reaction (RT-PCR) analysis, whereas protein expression was assessed by immunohistochemistry (IHC) and Western blot.

*GAPDH*, glyceraldehyde-3-phosphate dehydrogenase.

### Northern blot analysis

Total RNA (3–15 μg) was separated by electrophoresis on a 1% agarose gel and transferred to nylon membranes (Schleicher & Schuell BioScience, Keene, NH, USA) as previously described.^28^ The blot was hybridized with a 291-bp random-primed *SBEM*^32^P-labelled probe, generated as previously described.^29^ Equal loading and integrity of RNA were monitored by ethidium bromide staining of the 28s and 18s subunits of rRNA.

### Western blot analysis

Immunoblotting analysis of SBEM was performed using an SBEM monoclonal antibody H39C51 (Dr T. Colpitts, Abbott Laboratories), at a dilution of 1:500.^19^ Equal amounts of total protein lysates (25 μg) were extracted and analysed by sodium dodecyl sulphate–polyacrylamide gel electrophoresis as previously described,^26^ in the absence or presence of a SBEM neutralizing peptide.

### Immunohistochemistry

Serial sections (5 μm) of the TMAs were stained with the SBEM monoclonal antibody H218C31 at a dilution of 1:800 (Table 1). Commercially available antibodies were utilized for Ki67, CK5/6, epidermal growth factor receptor (EGFR) and HER-2 (Table 1), while psoriasin/S100A7 antibody has been previously validated by our group.^30^ IHC was performed as recently reported.^31^ Briefly, sections were dewaxed, rehydrated and then submitted to heat-induced antigen retrieval for 8 min in the presence of a citrate buffer (CC1; Ventana Medical Systems, Tucson, AZ, USA) using an automated tissue immunostainer (Discovery Staining Module; Ventana Medical Systems). The initial dilution quoted above was diluted further 1:3 with buffer dispensed onto the slide with the primary antibody. For each set of experiments, a strong SBEM+ and an SBEM– tumour sample, as previously assessed by RT-PCR, were included as controls. SBEM monoclonal antibody H218C31 was further validated at the IHC level, by pre-incubation for 4 h at 4°C with a 20-fold excess of a SBEM peptide (69–89aa, RKDIPVLPKWVGDLPNGRVC).

**Table 1:** Details of antibodies and experimental conditions used for immunohistochemistry in the present study

Biomarker	Antibody clone	Supplier	Dilution	Incubation	Method*
SBEM	H218C31	Dr T. Colpitts, Abbott Laboratories	1:800	1 h at 42°C	CC1
HER-2	CB11	NovocCastra, Newcastle, UK	1:50	1 h at 42°C	CC1
EGFR	3C6	Ventana Systems, Tucson, AZ, USA	Dispensed	30 min at 42°C	Protease 1**
CK5/6	D5/16134	Zymed, San Francisco, CA, USA	1:20	1 h at 42°C	CC1
KI67	MIB1	Dako, Mississauga, Canada	1:50	1 h at 42°C	CC1
ERα	6F11	NovoCastra	1:50	1 hr at 42°C	CC1

* Mild and standard cell conditioning, using CC1 antigen retrieval buffer (Ventana Medical Systems).

** Ventana Medical Systems using protease-1 enzyme for antigen retrieval.

SBEM, small breast epithelial mucin; EGFR, epidermal growth factor receptor; CK, cytokeratin; ER, oestrogen receptor.

### Quantification and cut-off selection

Immunopositivity for SBEM protein expression in the TMAs was assessed using semiquantitative scoring (H-scores). H-scores were derived from a semiquantitative assessment of both staining intensity (scale 0–3) and the percentage of positive cells (0–100%), which, when multiplied, generated a score ranging from 0 to 300. TMA immunohistochemistry was evaluated independently by three investigators (G.P.S., C.P., P.H.W.) and where discordance (i.e. different scores given by different investigators) was found (30 discordant cases, which were 10% of our entire cohort), cases were re-evaluated and a consensus reached. Primary categorical analysis was as follows: breast cancers were considered SBEM+ with an IHC score of >0. Only cytoplasmic reactivity was evaluated and scored. Also, since there is at present no relevant clinical cut-off point reported for SBEM in the literature, several cut-off points were tested for the IHC score, equivalent to an absence of staining, a 25th percentile and median IHC score values. Results reported in this study were based only on an IHC score of >0. Positivity for psoriasin/S100A7 (a marker that correlates with indicators of poor prognosis) and CK5/6 (one of the markers of basal phenotype) was set at >0, for Ki67 at >30 (corresponding to the median of the ER− TMA), whereas for EGFR and HER-2, only breast cancers that showed strong, intense membranous immunopositivity of IHC 3+ (>30% of invasive tumour cells) were considered positive, as reported, used and recommended by other groups.^32^–^34^

### Statistical analysis

Correlations were assessed by Spearman’s rank correlation test (*r*), whereas associations between SBEM and other clinicopathological variables were tested using contingency methods (Fisher’s exact test). Kruskal–Wallis test and Dunn’s method test (which compares all pairs of columns) were taken as correction for multiple analyses. Univariate survival analyses were performed using the log rank test to generate Kaplan–Meier curves. Overall survival (OS) was defined as the time from initial surgery to the date of death attributable to breast cancer only. Relapse-free survival (RFS) was defined as the time from initial surgery to the date of clinically documented local or distant disease recurrence or death attributed to breast cancer. Statistical analyses were carried out using GraphPad Prism 4.02 version (GraphPad, San Diego, CA, USA) and SPSS 12 for windows statistics software (SPSS Inc., Chicago, IL, USA).

## Results

### SBEM mRNA and protein expression in primary breast cancers

As a first step in assessing SBEM protein expression, we examined whether an association existed between RNA and protein levels in a small panel of 10 randomly selected primary breast cancers. Serial sections obtained from biopsy specimens were evaluated for gene expression by both RT-PCR and Northern blot analysis, whereas protein expression was assessed by IHC and Western blot analysis.

**Figure 1:**
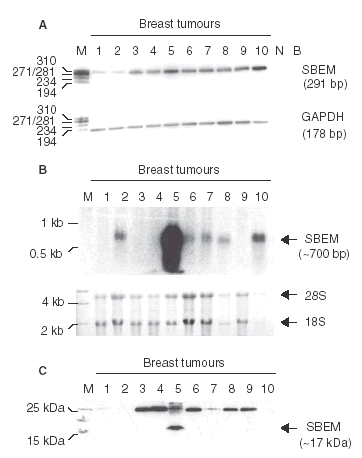
SBEM RNA and protein expression in a small panel of 10 human breast cancers. RNA and protein were extracted from breast cancers (*n* = 10) as described in Materials and Methods. Numbers (1–10) represent individual tumours. **A**, RT-PCR analysis. ‘N’ is a negative RT-PCR, i.e. reverse transcription performed minus MMLV enzyme, and ‘B’ is a PCR negative control. Lane M, PhiX174 RF DNA/Hae III DNA ladder. **B**, Northern blot analysis. Breast cancers were analysed as described in Materials and methods. Fifteen micrograms of total RNA from tumours 1–7, three from tumour no. 8, 10 μg from no. 9 and 6 μg from no. 10 were separated on agarose gels and hybridized using *SBEM*^32^P-labelled probes. Loading controls are provided with 18s and 28s RNA bands on the bottom panel M, ladder. **C**, Western blot analysis. Total proteins (25 μg) were analysed by sodium dodecyl sulphate–polyacrylamide gel electrophoresis using the SBEM H39C51 monoclonal antibody. M = ladder.

All breast cancers were positive by RT-PCR (10/10) and a single band of 291 bp corresponding to *SBEM* mRNA was detected (Figure 1A). Various levels of expression were observed (low to moderate in tumour samples 1, 3, 4 and 9; high in all others; Figure 1A, Table 2). Only tumours that showed the highest expression by RT-PCR were also positive by Northern blot analysis. Northern blot analysis showed a ∼700-bp *SBEM* transcript, which was identified in six out of 10 samples, with the highest level of expression observed in tumour sample no. 5 followed by tumour sample no. 10 (Figure 1B).

Western blot analysis revealed detectable levels of SBEM protein only in tumour sample no. 5 (which corresponded to the high *SBEM* mRNA expressor detected by Northern blot), whereas all others were negative (Figure 1C). In addition to the expected *SBEM* band, a 25-kDa band was sometimes apparent. However, only the ∼17-kDa band was related to SBEM protein, as repeatedly confirmed by peptide competition assay (data not shown).

**Figure 2:**
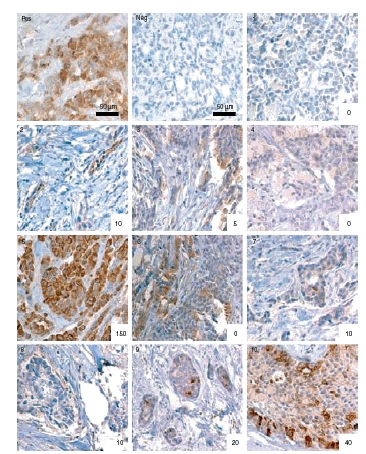
Immunohistochemical analysis of SBEM expression in the small panel of 10 human breast cancers. Immunohistochemistry was performed as described in Materials and Methods. Patient/sample identification is indicated on top left of each picture, and the corresponding H-score for IHC is presented on the bottom right corner. H-scores were derived from semiquantitative assessment of both staining intensity (scale 0–3) and the percentage of positive cells (0–100%) and, when multiplied, generate a score ranging from 0 to 300. Bar = 50 μm.

**Figure 3:**
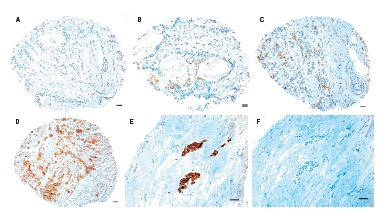
SBEM expression, determined by immunohistochemistry in ER+ and ER− tissue microarrays. **A**, ER+ cancer stained with the SBEM monoclonal antibody (H218C31) showing no expression (H-score of 0). **B**, ER+ tumour showing low expression (H-score of 30). **C**, ER+ breast cancer showing medium expression (H-score of 60). **D**, ER+ tumour showing high expression (H-score of 225). **E**, ER− breast cancer (ER and PR levels of 2.3 and 8.9 fmol/mg protein, respectively) immunoreactive for the H218C31 SBEM-specific antibody showing high expression. **F**, Serial section of the ER− breast cancer, where the primary antibody was incubated with ×20-fold excess SBEM peptide and abolished the specific stain.

Results from immunohistochemical analysis revealed that seven of the 10 breast cancers were immunopositive for SBEM (Table 2; Figure 2). Incubation with a 20-fold excess of the peptide abolished the SBEM signal (Figure 3E,F). The highest protein levels were detected in tumour no. 5, followed by tumour no. 10 (Table 2; Figure 2). No immunoreactivity was noted in tumours 1, 4 or 6. Notably, in tumours 4, 7 and 10, immunopositivity was observed in the surrounding normal mammary gland (data not shown), whereas in tumour no. 3, some normal breast ducts were SBEM+ (data not shown). Overall, SBEM protein expression (determined by IHC) correlated with *SBEM* gene expression [determined by RT-PCR (*r* = 0.727, *P* = 0.017 two-tailed, *n* = 10) and also by Northern blot analysis (tumours 5, 10, 8, 7 and 2; Table 2)].

### SBEM protein expression and clinicopathological prognostic variables in breast cancer cohort (*n* = 300)

**Figure 4:**
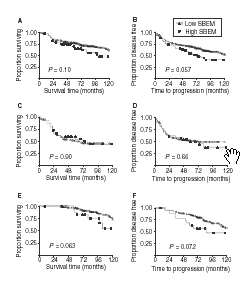
Kaplan–Meier graphs for overall survival and relapse free survival-time to progression for SBEM expression in the entire breast cancer cohort (**A,B**), ER α− (**C,D**) and ERα+ cancers (**E,F**). Symbols on the graph lines represent censored data, *P*-values are given for log rank tests. **A** (top left), *n* = 300; low SBEM events = 80, high SBEM events = 23; **B** (top right), *n* = 300; low SBEM events = 95, high SBEM events = 26; **C** (left), *n* = 149; low SBEM events = 57, high SBEM events = 16; **D** (right), *n* = 149; low SBEM events = 55, high SBEM events = 17; **E** (bottom left), *n* = 151; low SBEM events = 23, high SBEM events = 7; **F** (bottom right), *n* = 151; low SBEM events = 40, high SBEM events = 9.

Serial TMA sections were stained using IHC with specific antibodies recognizing SBEM, Ki67, CK5/6, EGFR, HER-2 and psoriasin/S100A7 (Table 1). Cytoplasmic reactivity was observed and scored for SBEM and positivity was defined as an IHC score of >0. From our studies, data on SBEM protein expression in 300 breast cases were obtained, but for the analysis of some of the other markers the numbers of cases analysed are slightly lower due to missing tumour cores on the TMAs, or unavailable data for analysis (Table 3). In this combined cohort of ER+ and ER− invasive breast cancers, 18% of tumours were positive for SBEM (*n* = 53/300). Using categorical analysis (i.e. SBEM defined as positive or negative), SBEM was strongly positively associated with psoriasin/S100A7 [Fisher’s exact test, *P* ≤ 0.0001, *n* = 280, odds ratio (OR) 6.77, 95% confidence interval (CI) 3.2, 14; Table 3], a marker that correlates with other indicators of poor prognosis,^30^,^35^,^36^ and negatively associated with Ki67, a marker of proliferation (Fisher’s exact test, *P* = 0.009, *n* = 230, OR 0.356, 95% CI 0.16, 0.78), and ERα (Fisher’s exact test, *P* = 0.049, *n* = 300, OR 0.536, 95% CI 0.29, 0.98). No other statistically significant associations between SBEM and established prognostic factors such as tumour size, axillary lymph node status and progesterone receptor were observed (Table 3). Interestingly, even though no association existed between overall survival and SBEM expression (Figure 4A), a trend toward significance was observed for those breast cancers that exhibited high levels of SBEM positivity and a shorter time to disease progression, compared with those that were SBEM− [low levels, *P* = 0.057, *n* = 300, hazard ratio (HR) 0.65, 95% CI of ratio 0.37, 1.04; Figure 4B]. This trend, along with the strong association observed with psoriasin expression, prompted us to examine further the relationship between SBEM protein expression and the ER status of the tumours.

**Table 3:** Distribution of SBEM+ and SBEM− cases within the cohort of 300 patients

Prognostic factor/ characteristics	Number (*n*)	Subgroup cut-offs	SBEM+	%	SBEM−	%	*P-*value
EGFR	248	+	6	14	23	11	0.61
			
		−	38	86	181	89	
HER-2	231	+	16	37	48	32	0.13
			
		−	27	67	140	68	
CK5/6	272	+	16	33	100	45	0.20
			
		−	32	67	124	55	
Ki67	230	x ≥ 30	9	20	78	42	**0.009**
			
		x < 30	35	80	108	58	
S100A7	280	+	37	77	77	33	**<0.0001**
			
		−	11	23	155	67	
PR (LBA)	300	x ≥ 10 fmol/mg	32	60	152	62	0.88
			
		x < 10 fmol/mg	21	40	95	38	
Node	300	+	17	32	55	22	0.16
			
		−	36	68	192	78	
Age	300	x > 50	45	85	185	75	0.15
			
		x≤50	8	15	62	25	
Grade	290	Low (3–5)	8	16	33	13	0.70
			
		Mod (6–7)	28	55	121	51	
			
		High (8–9)	15	29	85	36	
Size	300	>20 mm	35	66	172	70	0.62
			
		≤20 mm	18	34	75	30	
ERα (IHC)	300	+	20	38	131	53	**0.049**
			
		−	33	62	116	47	

For each characteristic, clinical and pathological (see Materials and Methods), the total number of cases is given (*n)*. *P*-values have been calculated following Fisher’s exact test. Cut-offs were previously set (Materials and Methods). Statistical analysis was performed with GraphPad Prism 4.02 software (San Diego, CA, USA), and statistically significant values are in bold.

EGFR, epidermal growth factor receptor; CK, cytokeratin; PR, progesterone receptor; LBA, ligand binding assay; ER, oestrogen receptor.

### SBEM protein expression, clinicopathological prognostic variables and survival in ER− breast cancers

In the ER− breast cancers analysed, only 22% of tumours were SBEM+ (*n* = 33/149). There was a positive and statistically significant correlation between SBEM and psoriasin/S100A7 expression (Spearman coefficient, *r* = 0.44, *P* < 0.0001, *n* = 146, 95% CI 0.29, 0.56). A significant inverse correlation with Ki67 (Spearman coefficient, *r* = −0.39, *P* < 0.0001, *n* = 148, 95% CI −0.52, −0.23) and CK5/6, a basal epithelial phenotype marker, was also observed (*r* = −0.34, *P* < 0.0001, *n* = 130, 95% CI −0.48, −0.17). Univariate analysis showed that SBEM was significantly associated with psoriasin/S100A7 (Fisher’s exact test, *P* = < 0.0001, *n* = 146, OR 14.48, 95% CI 3.3, 63.5; Table 4) and less significantly with HER-2 (Fisher’s exact test, *P* = 0.046, *n* = 135, OR 2.39, 95% CI 1.04, 5.48; Table 4). In the same cohort, SBEM was negatively associated with Ki67 and CK5/6, respectively (Fisher’s exact test, *P* = < 0.0001, *n* = 148, OR 0.232, 95% CI 0.098, 0.545; and *P =* < 0.0001, *n* = 130, OR 0.147, 95% CI 0.057, 0.381; Table 4), whereas lack of SBEM expression was associated with higher tumour grade (Fisher’s exact test, *P* = 0.0015, *n* = 139). No other statistically significant associations between SBEM and established prognostic factors such as tumour size, axillary lymph node status and PR were observed (Table 4). Furthermore, no difference in disease outcome (OS and RFS) was found between low and high SBEM expression (Figure 4C,D).

**Table 4:** Distribution of SBEM+ and SBEM− cases within the ER− cohort (*n* = 149)

Prognostic factor/ characteristics	Number (*n*)	Subgroup cut-offs	SBEM+	%	SBEM−	%	*P-*value
EGFR	116	+	5	20	23	25	0.79
			
		−	20	80	68	75	
HER-2	135	+	14	42	24	23	**0.046**
			
		−	19	58	78	77	
CK5/6	130	+	7	24	69	68	**<0.0001**
			
		−	22	76	32	32	
Ki67	148	x ≥ 30	9	27	71	62	**<0.0001**
			
		x < 30	24	73	44	38	
S100A7	146	+	30	94	58	51	**<0.0001**
			
		−	2	6	56	49	
PR (LBA)	149	x ≥ 10 fmol/mg	13	39	36	31	0.40
			
		x < 10 fmol/mg	20	61	80	69	
Node	149	+	17	52	55	47	0.70
			
		−	16	48	61	53	
Age	149	x > 50	25	76	69	59	0.10
			
		x≤50	8	24	47	41	
Grade	139	Low (3–5)	3	10	3	3	**0.015**
			
		Mod (6–7)	15	48	32	30	
			
		High (8–9)	13	42	73	67	
Size	149	>20 mm	23	70	82	71	1.0
			
		≤20 mm	10	30	34	29	

For each clinical and pathological characteristic (see Materials and Methods), the total number of cases is given (*n*). *P*-values have been calculated following Fisher’s exact test. Cut-offs were previously set (Materials and Methods). Statistical analysis was performed with GraphPad Prism 4.02 software (San Diego, CA, USA), and statistically significant values are in bold.

EGFR, epidermal growth factor receptor; CK, cytokeratin; PR, progesterone receptor; LBA, ligand binding assay.

### SBEM protein expression, clinicopathological prognostic variables and survival in ER+ breast cancers

In the ER+ breast cancers, 13% of tumours (*n* = 20/151) were immunopositive for SBEM (Figure 3A–D). SBEM was significantly correlated and associated with psoriasin/S100A7, respectively (Spearman’s coefficient, *r* = 0.21, *P* = 0.015, *n* = 132, 95% CI 0.036, 0.373; and Fisher’s exact test, *P* = 0.016, *n* = 134, OR 4.053, 95% CI 1.34, 12.21; Table 5). No other associations between SBEM and established prognostic factors were observed in this better prognosis cohort (Table 5). However, using the log rank test to generate Kaplan–Meier curves, an interesting trend toward significance was observed in disease outcome (OS and RFS), with patients with higher SBEM levels being associated with worse survival and a shorter time to progression (*P* = 0.063, HR 0.461, 95% CI of ratio 0.117, 1.060; and *P* = 0.072, HR 0.522, 95% CI of ratio 0.172, 1.078, respectively; Figure 4E,F).

**Table 5:** Distribution of SBEM+ and SBEM− cases within the ER-positive cohort (*n* = 151)

Prognostic factor/ characteristics	Number (*n*)	Subgroup cut-offs	SBEM+	%	SBEM−	%	*P-*value
EGFR	132	+	1	5	0	0	0.14
			
		−	18	95	113	100	
HER-2	96	+	2	20	24	28	0.72
			
		−	8	80	62	72	
CK5/6	142	+	9	47	31	25	0.06
			
		−	10	53	92	75	
Ki67	82	x ≥ 30	0	0	7	10	0.58
			
		x < 30	11	100	64	90	
S100A7	134	+	7	44	19	16	**0.016**
			
		−	9	56	99	84	
PR (LBA)	151	x ≥ 10 fmol/mg	19	95	116	89	0.70
			
		x < 10 fmol/mg	1	5	15	11	
Node	151	+	0	0	0	0	1.0
			
		−	20	100	131	100	
Age	151	x > 50	20	100	116	89	0.22
			
		x≤50	0	0	15	11	
Grade	151	Low (3–5)	5	25	30	23	0.96
			
		Mod (6–7)	13	65	89	68	
			
		High (8–9)	2	10	12	9	
Size	151	>2	12	60	90	69	0.45
			
		≤2	8	40	41	31	

For each clinical and pathological characteristic (Materials and Methods), the total number of cases is given (*n*). *P*-values have been calculated following Fisher’s exact test. Cut-offs were previously set (Materials and Methods). Statistical analysis was performed using the GraphPad Prism software version 4.02 (San Diego, CA, USA), and statistically significant values are in bold.

EGFR, epidermal growth factor receptor; CK, cytokeratin; PR, progesterone receptor; LBA, ligand binding assay.

### SBEM protein expression in normal breast tissue

IHC was also performed on a TMA consisting of 68 normal tissues (Biomax; cat. No. BN08013). Moderate to strong immunoreactivity for SBEM was detected in nine normal tissues (three breast ducts, six fibro-fatty tissues) and SBEM expression was calculated at 13% (9/68 cases). Three malignant breast biopsy specimens, included as markers for orientation purposes in the TMA, showed no specific SBEM reactivity (0/3). A highly positive SBEM breast cancer sample and a SBEM− one, previously assessed by RT-PCR, were used as controls, for IHC analysis.

## Discussion

Most data on SBEM thus far have been accumulated from gene expression studies, whereas SBEM protein expression in human breast cancers has not been explored. As a first step in addressing SBEM protein expression in breast cancer, we examined the relationship between gene and protein expression. As expected, differences were observed when using techniques with known different levels of sensitivity. For example, when all samples were positive using RT-PCR, SBEM protein was detected in only one tumour by Western blot analysis. This underscores the critical importance of setting standards and references to be used in the assessment of expression of a specific biomarker for clinical management purposes. A significant correlation was found between results from protein assessment by IHC and RNA measurement by semiquantitative RT-PCR. This finding was surprising, as even though IHC is currently used to assess biomarker positivity (ER, for example), we were not expecting statistical significance when comparing with a semiquantitative assay performed on such a small cohort (*n* = 10). This suggests that a strong association exists between SBEM RNA and protein expression. It also suggests that an even better estimation of the expression of this gene might be performed with the more quantitative real-time PCR. If SBEM is to be used as a clinical biomarker, the possibility of accurately measuring its levels from a tiny amount of tissue (biopsy specimen) is another major advantage to be considered.

The findings presented here include the largest number of breast cancers to date to examine SBEM protein expression. In a combined cohort of 300 ER+ and ER− invasive breast cancers, it was found that 18% of cancers were positive for SBEM. SBEM was also found to be highly associated with psoriasin/S100A7, a member of the S100 family of genes. Psoriasin/S100A7 is known to correlate with indicators of poor prognosis, specifically in ERα− breast cancers, and may represent an independent prognostic factor for clinical outcome.^30^,^35^,^36^ To investigate SBEM protein expression further in separate subgroups, we divided the patient cohort according to ER status (as determined by both the ligand binding assay and IHC). In the ERα− TMA, which represents a more aggressive group of breast cancers, SBEM protein was detected in 22% of breast cancers. Univariate analysis showed that SBEM was strongly associated with psoriasin/S100A7 and less so with HER-2, the latter being well documented to confer poor prognosis in breast cancer patients. Interestingly, a significant association between HER-2 determined by IHC and increasing amounts of SBEM mRNA (*P* = 0.003) has also been reported by others.^37^ These results suggest that SBEM, like psoriasin/S100A7 and HER-2, may also be associated with poor prognosis, an observation which is consistent with our previous findings that SBEM mRNA expression was higher in node-positive tumours.^20^ Interestingly, in the ER-positive/node-negative cohort, SBEM expression was also significantly associated with psoriasin/S100A7, and perhaps identifies, within a breast cancer cohort generally considered to have a very good prognosis, a minority of patients more likely to experience recurrence of disease. This is emphasized by the fact that strong trends were observed toward both a lower survival rate and a higher recurrence of the disease in SBEM+ patients. SBEM protein, however, was more frequently expressed in ER− breast cancers, consistent with its positive association with psoriasin/S100A7 and HER-2. Furthermore, it is interesting that a SBEM correlation exists with Ki67, but not with tumour grade. However, correlation with proliferation index but not with grade could arise from lack of association or competing association with the other components of tumour grade (which was based on the Nottingham grading score, in which the grade reflects nuclear morphology and tubular differentiation, as well as proliferation rate). Further pathological analysis is required to explore this aspect.

Because of the heterogeneous nature of breast cancers, it is unlikely that any single tumour marker would be sufficient for breast cancer diagnosis or detection of metastasis. Indeed, an increasing number of reports have suggested that the use of multiple markers could indeed improve the likelihood of detecting tumour cells across the population.^23^,^38^,^39^ Identification of a new subset of patients using SBEM protein detection would be likely to enhance the potency of such a multimarker set. In line with such a multimarker strategy to disease management, we have previously shown that both SBEM and mammaglobin gene expression correlate positively with axillary lymph node metastasis.^20^ SBEM has also been included in a list of markers (18 in total) currently used for the detection of disseminated tumour cells, and their significance and limitations have been reviewed and discussed.^22^ In addition, a marker set containing CK19, p1B, EGP2 and SBEM has been reported to facilitate the discrimination between negative and positive lymph nodes in breast cancer patients.^23^ Recently, *SBEM* (BS106) gene expression has also been investigated with real-time PCR in a series of breast cancers, lymph nodes from cancer and non-cancer patients and normal breast tissues.^37^*SBEM* was highly expressed in all but one breast cancers and in all normal breast tissues. The authors concluded that SBEM added to the potential utility of other markers in breast cancer, such as mammaglobin and CK19.^37^

The TMAs used in this study were constructed with duplicate cores (0.6 mm) derived from each tumour sample. Our results have shown some heterogeneity in SBEM staining. Furthermore, it may be a matter of concern whether two cores are entirely representative of gene expression in a large tumour sample, as they could result in both over- and underscoring of protein expression in the TMA. However, although we cannot entirely exclude this possibility, previous studies by others have shown that with use of the standard breast cancer prognostic markers (ER, PR and HER-2), two cores are sufficient and have resulted in >95% accuracy.^40^–^42^ Similarly, two cores have also been reported to provide a high level of accuracy in the TMA analysis of other cancers, such as metastatic prostate and ovarian cancer.^43^,^44^

Overall, SBEM expression was observed in 22% of ERα− and 13% of ERα+ breast cancers. No statistically significant association between SBEM expression and clinical outcome was observed. However, in ER+ breast cancers a definite trend toward significance between SBEM protein levels and disease outcome of patients was observed, suggesting that SBEM may be associated with worse survival and shorter time to progression in this specific subgroup. In ER− cancers, SBEM protein expression was highly associated with some markers of poor prognosis. We believe further analyses are needed, using real-time RT-PCR, to refine our assessment of SBEM expression in breast cancers. Altogether, these results suggest that SBEM may identify a unique subset of breast cancers and may have future implications for therapeutic management of the disease.

## Abbreviations:

CI: confidence interval
CK: cytokeratin
EGFR: epidermal growth factor receptor
EGP: epithelial glycoprotein
ER: oestrogen receptor
GAPDH: glyceraldehyde-3-phosphate dehydrogenase
HR: hazard ratio
IHC: immunohistochemistry
LBA: ligand binding assay
MBTB: Manitoba Breast Tumour Bank
MMLV: Moloney murine leukaemia virus
OR: odds ratio
OS: overall survival
PR: progesterone receptor
RFS: relapse-free survival; RT-PCR, reverse transcriptase-polymerase chain reaction
SBEM: small breast epithelial mucin
TMA: tissue microarray
